# Plasma Proteomic Changes in *GRN* and *C9orf72* Frontotemporal Dementia

**DOI:** 10.1111/ene.70704

**Published:** 2026-07-12

**Authors:** Joel Simrén, Andrea L. Benedet, Guglielmo Di Molfetta, Ilaria Pola, Kübra Tan, Valentina Cantoni, Ilenia Libri, Jasmine Rivolta, Roberta Ghidoni, Henrik Zetterberg, Barbara Borroni, Nicholas J. Ashton

**Affiliations:** ^1^ Department of Psychiatry and Neurochemistry Institute of Neuroscience and Physiology, The Sahlgrenska Academy at the University of Gothenburg Gothenburg Sweden; ^2^ Clinical Neurochemistry Laboratory Sahlgrenska University Hospital Mölndal Sweden; ^3^ Department of Clinical and Experimental Sciences University of Brescia Brescia Italy; ^4^ Molecular Markers Laboratory, IRCCS Istituto Centro San Giovanni di Dio Fatebenefratelli Brescia Italy; ^5^ Department of Neurodegenerative Disease Institute of Neurology, UCL London UK; ^6^ UK Dementia Research Institute, UCL London UK; ^7^ Hong Kong Center for Neurodegenerative Diseases Hong Kong China; ^8^ Wisconsin Alzheimer's Disease Research Center University of Wisconsin School of Medicine and Public Health Madison Wisconsin USA; ^9^ Department of Old Age Psychiatry Institute of Psychiatry, Psychology & Neuroscience, King's College London London UK; ^10^ Banner Alzheimer's Institute Phoenix Arizona USA; ^11^ Banner Sun Health Research Institute Sun City Arizona USA

**Keywords:** biomarkers, frontotemporal dementia, proteomics

## Abstract

**Background:**

Biomarkers reflecting the complex pathophysiology of genetic frontotemporal dementia (FTD) will be increasingly important with the advent of therapeutic trials aiming to slow or prevent the disease. In this study, we aimed to identify blood biomarker candidates using a multiplex panel of CNS‐related proteins.

**Methods:**

We cross‐sectionally evaluated 67 carriers (21 presymptomatic and 46 symptomatic) of pathogenic FTD‐causing mutations in the *GRN* (*n* = 30 symptomatic) and *C9orf72* (*n* = 16 symptomatic) genes and 42 matched non‐carriers. Clinical severity was estimated using the CDR Dementia Staging Instrument with National Alzheimer Coordinating Centre Frontotemporal Lobar Degeneration component (CDR plus NACC FTLD). A total of 124 CNS‐related proteins were measured in plasma using the NUcleic acid Linked Immuno‐Sandwich Assay (NULISA) CNS panel. Group‐level changes were then investigated using linear and non‐linear regression models.

**Results:**

In *GRN‐* and *C9orf72*‐FTD, neurofilament light (NfL) was the most clearly altered protein compared with non‐carriers (*GRN:* β [95% CI] = 4.0 standard deviations [3.6–4.4], *C9orf72*: β = 2.8 [2.2–3.4]), followed by neurofilament heavy (NfH; *GRN*: β = 0.83 [0.39–1.3], *C9orf72*: β = 1.4 [0.8–2.0]). Proteins exclusively altered in *GRN*‐FTD included glial fibrillary acidic protein (GFAp; β = 0.50 [0.20–0.81]) and vascular cell adhesion protein 1 (VCAM1; Standardized β = −0.90 [−1.4 to −0.38]), changing with increasing disease severity. Neuronal pentraxin receptor (NPTXR; β = −0.94 [−1.5 to −0.4]) was selectively reduced in *C9orf72‐*FTD. Nominally changed proteins in *C9orf72*‐FTD included several inflammatory mediators.

**Conclusions:**

Using this multiplex panel, established markers recapitulated previously established trends, while less‐studied biomarker candidates were also identified. If validated in independent cohorts, these candidates could broaden the repertoire of blood biomarkers reflecting genetic FTD pathophysiology.

## Graphical abstract

Multiplex plasma profiling of 124 CNS‐related proteins in genetic frontotemporal dementia (FTD) identified NfL and NfH as the most consistently altered biomarkers in both GRN‐ and C9orf72‐associated disease. Distinct protein changes emerged across genotypes, including changes in GFAP and VCAM1 in GRN‐FTD, and reduced NPTXR as well as nominally altered inflammatory proteins in C9orf72‐FTD. These findings reinforce established markers while highlighting additional candidates that may capture complementary aspects of genetic FTD pathophysiology and disease progression.

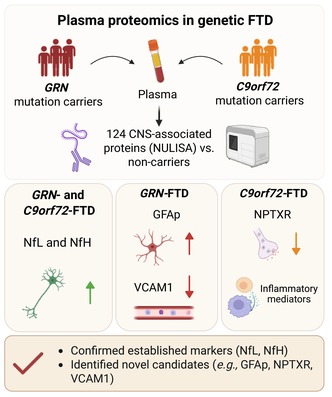

## 1 Introduction

Frontotemporal dementia (FTD) is one of the most common causes of early onset dementia [1]. Development and regulatory approval of putatively disease‐modifying drugs for Alzheimer's disease (AD) have been greatly aided by the development of biomarkers reflecting key pathologies of the disease [2]. Conversely, the lack of such markers for FTD likely limits progress in the quest for effective therapies for these disorders. Due to the predictable TAR DNA binding protein 43 kDa (TDP‐43) pathology in the two most common causes of genetic FTD—*GRN* and *C9orf72* mutations—they present compelling models for biomarker development and studies of mechanisms underlying the disease [3, 4]. Recent proteomics studies in cerebrospinal fluid have found both shared and distinct alterations in these two causes of genetic FTD [5, 6]. Due to the invasiveness of a lumbar puncture, identifying proteins measurable in plasma that reflect key pathophysiological alterations will be helpful as response markers in clinical trials, and to corroborate phenotypes found in preclinical model systems. There is still scarce data on proteomic changes in plasma of individuals with FTD, and the studies that have been published to date have found changes of unknown significance in mixed FTD populations [7], or did not find any changes between genetic FTD subtypes and healthy controls [8]. Recently, we reported promising findings from a pilot study of individuals with GRN‐FTD using a novel NUcleic acid Linked Immuno‐Sandwich Assay (NULISA) multiplex plasma panel targeting proteins related to neurodegenerative diseases [9]. The analytical sensitivity of the NULISA platform along with the careful selection of proteins enriched in CNS or previously associated with brain disorders provides an opportunity to discover novel biomarkers for this group of disorders. In the present exploratory study, we expanded our pilot study to include a larger number of symptomatic *GRN* carriers, as well as symptomatic and presymptomatic carriers of pathogenic *C9orf72* and *GRN* mutations, to uncover biomarker candidates distinct or shared between these genetic causes of frontotemporal lobar degeneration (FTLD) with TDP‐43 and to determine whether proteomic changes are present before symptom onset. This enabled us to investigate biomarker candidates at different stages of the disease that may contribute to understanding mechanisms involved in disease progression, improving diagnostics, and drug development.

## 2 Methods

### 2.1 Participants

This retrospective cross‐sectional case–control study enrolled participants at the Center for Neurodegenerative Disorders, University of Brescia, Italy. No preregistration of the study has been done. Presence of pathogenetic *GRN* or *C9orf72* mutations was established according to procedures that have been described elsewhere [10]. No sample size calculations were performed but were determined based on the availability of plasma samples, with group sample size being similar to previous proteomic studies in genetic FTD. Presymptomatic mutation carriers and non‐carriers, recruited among spouses or patients' family members, were also included. Symptomatic individuals met international criteria for each clinical FTD syndrome [11, 12, 13, 14]. The CDR Dementia Staging Instrument with National Alzheimer Coordinating Centre FTLD component (CDR plus NACC FTLD, abbreviated as CDR‐FTLD) was used to assess disease severity [15]. Presymptomatic *C9orf72* carriers (*n* = 3) were not included in subsequent statistical analyses but are shown in plots. 18 *GRN*‐FTD, 2 presymptomatic *GRN* carriers and 20 non‐carriers included here were part of a previous pilot study [9]. All participants gave written informed consent according to the Declaration of Helsinki. The local Ethics Committee (NP 2189) approved the study.

### 2.2 Biomarker Measurements

Blood was collected by venipuncture in EDTA tubes, centrifuged and stored at −80° until analysis. NULISA assays were performed at the Clinical Neurochemistry Laboratory, University of Gothenburg, Mölndal, Sweden. Samples were analyzed with the CNS Disease Panel (124 targets listed in Table S1; Alamar Biosciences, Fremont, CA, USA) on an ARGO HT system, and the resulting DNA reporters were subsequently sequenced, processed and normalized as described in more detail previously [16]. Log_2_‐transformation was then applied to obtain NULISA Protein Quantification (NPQ) units for downstream statistical analysis. Limit of detection (LOD) was defined as three standard deviations above the blank. Analytes detectable in < 50% of participants were excluded. These included ubiquitin C‐terminal hydrolase L1 (2.5% detectability), pleiotrophin (0%), β‐synuclein (24%), phosphorylated TDP‐43 at amino acid 409 (pTDP‐43_409_; 35%), phosphorylated tau 217 (39%) and calretinin (49%). The E4 isoform of apolipoprotein E (apoE) was detected in 39%, with detectability being consistent with the presence of at least one *APOE* ε4 allele [17]. 117 proteins were detectable in > 70% of samples (Table S1). NPQ values for all analytes stratified by group can be found in Table S2.

### 2.3 Statistical Analysis

Prior to the statistical analysis presented in volcano plots, biomarker NPQ values were standardized based on the mean and standard deviation of non‐carriers. No imputation of missing values or outlier removal was performed. Groups were compared using linear regression models including group, age and sex as predictor variables and biomarker as outcome. 95% confidence intervals (CI) of the standardized β coefficients were obtained using the *confint* function. To visualize biomarkers across the disease continuum, we fitted locally estimated scatterplot smoothing (LOESS) line, which is a flexible nonlinear regression approach. Using this approach, biomarker targets identified as significantly different between *GRN‐*FTD versus non‐carriers were modeled against the CDR‐FTLD (treated as a continuous variable; 1 = non‐carrier, 2 = presymptomatic carrier, 3 = CDR‐FTLD of 0.5 + symptoms, 4 = CDR‐FTLD of 1, 5 = CDR‐FTLD of 2, 6 = CDR‐FTLD of 3). Unadjusted *p*‐values (*P*_unadjust_) are reported in volcano plots to display proteins that may be of interest; only proteins with two‐sided *p* < 0.05 adjusted for false discovery rate (FDR; denoted *P*_adjust_) were considered statistically significant. All statistical analyses and visualizations were performed in *R* (version 4.4.3). ChatGPT was used to generate code for linear regression analyses.

## 3 Results

The study cohort consisted of 42 non‐carriers, 3 presymptomatic *C9orf72* mutation carriers, 18 presymptomatic *GRN* mutation carriers, 16 symptomatic *C9orf72* mutation carriers, and 30 symptomatic *GRN* mutation carriers. Participants' characteristics can be found in Table 1.

**TABLE 1:** Participants characteristics.

	Non‐carrier	Presymptomatic *C9ORF72*	Presymptomatic *GRN*	Symptomatic *C9ORF72*	Symptomatic *GRN*
	*N* = 42^a^	*N* = 3^a^	*N* = 18^a^	*N* = 16^a^	*N* = 30^a^
Age	58 (47, 69)	60 (44, 63)	56 (50, 61)	65 (58, 71)	61 (56, 70)
Sex					
Female	26 (62%)	1 (33%)	12 (67%)	6 (38%)	19 (63%)
Diagnosis					
nfPPA				2 (13%)	18 (60%)
bvFTD				12 (75%)	9 (30%)
Other^b^				2 (13%)	3 (10%)
CDR plus NACC FTLD					
0	42 (100%)	3 (100%)	16 (89%)	0 (0%)	0 (0%)
0.5	0 (0%)	0 (0%)	2 (11%)	6 (38%)	3 (10%)
1	0 (0%)	0 (0%)	0 (0%)	7 (44%)	15 (50%)
2	0 (0%)	0 (0%)	0 (0%)	1 (6.3%)	8 (27%)
3	0 (0%)	0 (0%)	0 (0%)	2 (13%)	4 (13%)

Abbreviations: bvFTD, behavioral variant frontotemporal dementia; CBS, corticobasal syndrome; CDR plus NACC FTLD, clinical dementia rating plus national coordinating center frontotemporal lobar degeneration; FTD‐MND, frontotemporal dementia motor neuron disease; nfPPA, non‐fluent primary progressive dementia; PCA, posterior cortical atrophy.

^a^ Median (Q1, Q3); *n* (%).

^b^ CBS, FTD‐MND, PCA or svPPA.

**FIGURE 1:**
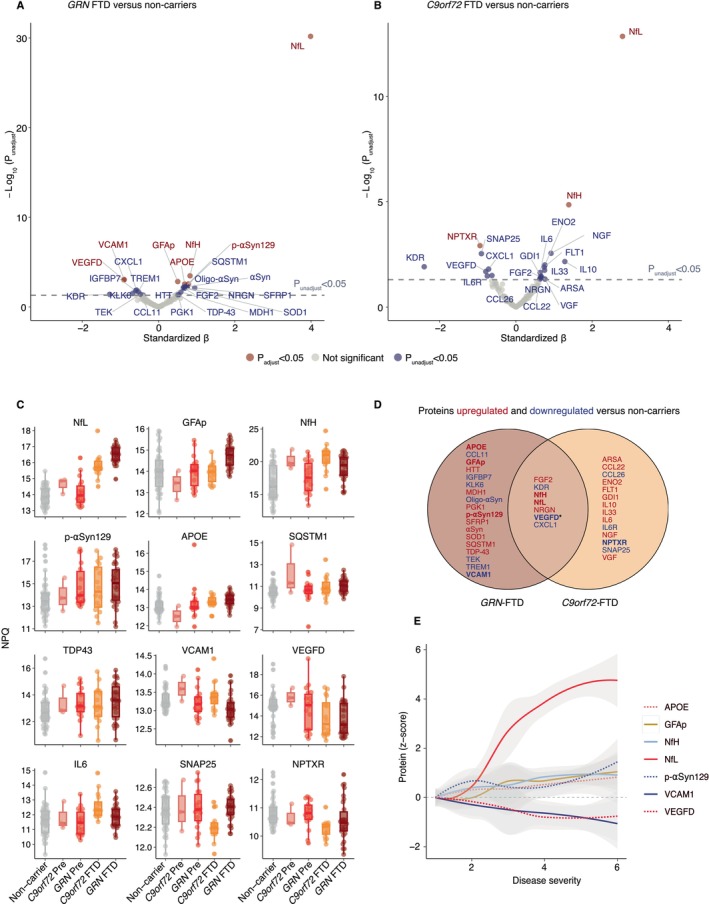
Plasma proteomic similarities and differences in genetic FTD. The volcano plots display proteins significantly changed (*P*_adjust_ < 0.05; red) and with *P*_unadjusted_ < 0.05 (blue) in *GRN* (A) and *C9orf72* frontotemporal dementia (FTD) (B) compared with non‐carriers, with standardized β for each biomarker target in linear models adjusting for age and sex. Note the different *y*‐axes in panel A and B. The boxplots (C) show NUcleic acid Linked Immuno‐Sandwich Assay (NULISA) protein quantification (NPQ) values for biomarkers significantly changed in symptomatic carriers in comparison with the other study groups. The boxes represent median and interquartile range, and whiskers reflect upper or lower quartile ±1.5 times the interquartile range. The Venn diagram (D) illustrates proteins nominally changed (*P*_unadjust_) when comparing *GRN‐* and *C9orf72*‐FTD versus non‐carriers. Proteins in bold highlight *P*_adjust_ < 0.05. Blue text reflects downregulated proteins and red text highlights upregulated proteins. Panel E visualizes biomarkers significantly changed in *GRN*‐FTD versus non‐carriers using a locally estimated scatterplot smoothing (LOESS) curve that models z‐scores in relation to increasing clinical disease severity in *GRN* carriers, with non‐carriers being used as a reference group. *P_adjust_ < 0.05 only in *GRN*‐FTD.

When comparing *GRN*‐FTD with non‐carriers, seven proteins were significantly changed (all *P*_adjust_ < 0.05; Figure 1A, exact *p*‐values in Table S3), including neurofilament light (NfL; β [95% CI] = 4.0 [3.6–4.4]), neurofilament heavy (NfH; β = 0.83 [0.39–1.3]), vascular endothelial growth factor‐D (VEGFD; β = −0.91 [−1.4 to −0.38]), vascular cell adhesion protein‐1 (VCAM1; β = −0.90 [−1.4 to −0.38]), glial fibrillary acidic protein (GFAp; β = 0.50 [0.20–0.81]), apolipoprotein E (apoE; β = 0.69 [0.25–1.1]) and phosphorylated α‐synuclein 129 (p‐αSyn129; β = 0.79 [0.20–0.81]). Another 18 proteins had *P*_unadjust_ < 0.05 (Figure 1A), with notable examples including TDP‐43 (β = 0.53 [0.02–1.05]) and sequestosome‐1 (SQSTM1; β = 0.71 [0.22–1.2]). Although detectability was only 35% for p‐TDP43_409_, we evaluated if there were any group‐level differences in the raw NPQ values. However, no significant changes were seen (all *p* > 0.05 vs. non‐carriers).

NfL (β = 2.8 [2.2–3.4]) and NfH (β = 1.4 [0.8–2.0]) were higher also in *C9orf72‐*FTD compared with non‐carriers (Figure 1B; Table S4). In addition, neuronal pentraxin receptor (NPTXR; β = −0.94 [−1.5 to −0.4]) was lower in *C9orf72*‐FTD vs. non‐carriers (Figure 1B). Another 18 proteins had *P*_unadjust_ < 0.05 (Figure 1B), including several chemokines and interleukins (e.g., IL6; β = 0.64 [0.09–1.2]). Boxplots of biomarkers of interest are shown in Figure 1C. The trend of a more prominent alteration in inflammatory mediators in *C9orf72*‐FTD was also evident when comparing the nominally changed proteins that overlapped in *GRN*‐ and *C9orf72*‐FTD versus non‐carriers (Figure 1D).

When instead directly comparing symptomatic mutation carrier groups, GFAp (β = 0.80 [0.46–1.1]), synaptosomal‐associated protein‐25 (SNAP25; β = 0.90 [0.44–1.4]) and NfL (β = 1.2 [0.58–1.9]) were higher in *GRN*‐ compared with *C9orf72*‐FTD. None of the inflammatory mediators passed correction for multiple testing, but several had *P*_unadjust_ < 0.05 (Table S5). Interestingly, although NfL was highest in *GRN*‐FTD, NfH was nominally higher in *C9orf72*‐FTD (*P*_unadjust_ < 0.05; β = 0.52 [0.05–1.0] vs. *GRN*‐FTD).

In presymptomatic *GRN*, no proteins were significantly changed vs. non‐carriers, although 15 proteins had *P*_unadjust_ < 0.05 (Table S6). We then modeled the proteins significantly altered in *GRN*‐FTD across the disease continuum. We found a sharp increase in NfL with increasing disease severity. Although less prominent, gradual increases were seen also for GFAp, NfH, p‐αSyn129 (Figure 1E), whereas changes in the opposite direction were seen for VCAM1 and VEGFD (Figure 1E).

## 4 Discussion

In this exploratory cross‐sectional study that investigated a panel of more than 100 proteins detectable in plasma from carriers of pathogenic FTD‐causing mutations, we found that NfL increases with greater disease severity, which is also seen when measured using single‐plex assays [18]. We also confirm that plasma GFAp is altered in *GRN*‐FTD, but not *C9orf72*‐FTD [19], or sporadic FTD without further specification on the underlying cause [20]. Beyond these markers, we found divergent patterns of less explored markers in genetic FTD, which reflect changes in biologically plausible cellular functions. Conversely, previous studies have found limited [8], or no changes in plasma of patients with FTLD spectrum disease [7]. This may reflect the superior sensitivity of the NULISA platform compared with proteomic methods used in previous exploratory studies, including proximity extension assays [16]. By combining a two‐step capture‐recapture immuno‐sandwich approach with DNA‐based signal amplification to minimize background noise, NULISA achieves ultrasensitive protein detection beyond that of most current proteomic platforms. The combination of analytical sensitivity allowing for biomarker discovery while including established biomarkers constitutes a strength of this novel platform.

This is reflected in the ability to robustly quantify plasma NfL, which is emerging as an important tool in the armamentarium of FTD‐related biomarkers, and the increases seen in the presymptomatic and early symptomatic phase of genetic FTD [21] supports its use to estimate disease stage and potentially also as a response marker in trials, as seen with tofersen in *SOD1*‐ALS [22]. The finding that *GRN‐*FTD had the highest NfL concentrations is consistent with previous studies in both CSF and plasma [5, 18, 21]. The elevated plasma NfH concentrations observed in both *C9orf72*‐FTD and *GRN*‐FTD in the present study, as well as previously reported increases in CSF, likely also reflect disease severity and neuroaxonal damage [23]. Notably, plasma phosphorylated NfH (pNfH) has been shown to increase near symptom onset in genetic FTD, suggesting that it may provide additional value beyond NfL as a marker of disease proximity [24]. In the present study, cross‐sectional modeling further supported the notion that NfL and NfH may provide complementary information for disease staging, although longitudinal studies are better suited to address this question [24]. Consistent with the notion that NfL and NfH may capture partly distinct aspects of disease biology, NfH concentrations were nominally higher in *C9orf72*‐FTD than in *GRN*‐FTD (*P*_unadjust_ < 0.05), despite the latter showing the highest NfL levels. This observation is in line with reports of particularly high pNfH concentrations in ALS [25] and may reflect the well‐established biological and clinical overlap between *C9orf72*‐associated FTD and ALS.

Plasma GFAp was exclusively increased in *GRN‐*FTD, which aligns well with previous data, demonstrating normal concentrations in other causes of genetic FTD (e.g., *C9orf72* and *MAPT*) [19, 26]. This finding speaks to the importance of conducting biomarker studies in stratified patient populations, as GFAp was unchanged in sporadic FTD in a recent study also employing the NULISA technique by Durcan et al. [20] In AD, where plasma GFAp is also increased, it is thought to reflect astroglial responses to extracellular amyloid plaques [27], whereas the increase seen in *GRN*‐FTD may be due to a hyperactivated microglia phenotype caused by progranulin haploinsufficiency, in turn causing astrogliosis [28]. This is also a possible cause of increased plasma apoE in *GRN‐*FTD carriers [28], as apoE is highly expressed in *GRN* knockout microglia [29]. Conversely, the finding that several inflammatory mediators were nominally altered in *C9orf72*‐FTD both versus non‐carriers and *GRN‐*FTD was surprising, given the role of progranulin in the regulation of inflammatory responses. However, *C9orf72* loss‐of‐function leads to pro‐inflammatory microglial and macrophage phenotypes in mice, which may explain the findings in this study [30]. A previous investigation examining similar proteins in *C9orf72* mutation carriers using an alternative analytical approach reported no alterations but identified several dysregulated inflammatory mediators in *GRN* and *MAPT*‐associated FTD [31]. Collectively, these discrepancies likely reflect methodological differences and variability in disease stage among cohorts.

Further, the trend of an increase (*P*_unadjusted_ < 0.05) in SQSTM1 (also termed p62; an autophagy‐related protein), may reflect a failing endolysosomal system in *GRN‐*FTD [28]. SQSTM1 was also nominally increased in plasma of individuals with sporadic FTD, and significantly in progressive supranuclear palsy in the study by Durcan et al. [20]. Interest in SQSTM1 stems from its role in the degradation of misfolded proteins [32]. Moreover, mutations in SQSTM1 have been found to as a rare cause of frontotemporal dementia [33]. Proteins that decreased included VCAM1 and VEGFD, possibly reflecting vascular pathology, as for example, VCAM1 regulates adhesion of immune cells to the endothelial wall, and progranulin has been suggested to be involved in vascular homeostasis [34]. However, the inflammatory environment created by progranulin haploinsufficiency may also be present in peripheral organs, meaning that it is unclear if these changes reflect CNS pathology or peripheral effects of insufficient progranulin.

NPTXR, involved in regulation of glutamatergic synapses, was decreased in *C9orf72*‐FTD, reflecting findings previously observed in CSF across several neurodegenerative diseases. Prior studies have shown reduced CSF NPTXR both in *C9orf72* and *GRN* carriers, although with a stronger effect in *C9orf72* [5, 6]. In our study, the reduction reached significance only in *C9orf72*‐FTD, while the non‐significant change in *GRN*‐FTD was in the same expected direction, suggesting that NPTXR may be more dysregulated in *C9orf72*‐associated disease. The clearer reduction of plasma NPTXR in *C9orf72* carriers may also explain why Durcan et al. did not find a similar change in unselected FTD patients. These findings support the potential of plasma NPTXs as disease‐related and possibly prognostic biomarkers [35], particularly given their proposed interaction with TDP‐43 [36]. Conversely, p‐αSyn129 was increased in *GRN*‐FTD. Due to the role of α‐synuclein in the presynapse, it may reflect synaptic dysfunction unrelated to α‐synuclein aggregates, although this needs to be studied further. P‐αSyn129 was also increased in sporadic FTD in the recent study by Durcan et al., which also employed the NULISA technique [20].

Finally, although TDP‐43 was nominally increased in *GRN*‐FTD but unchanged in *C9orf72*‐FTD. Phosphorylated TDP‐43_409_ showed low detectability (>limit of detection in only 35%) and no group differences. Although elevated pTDP‐43 has been reported in TDP‐43–associated FTD [37], its broad expression across extracerebral tissues (http://proteinatlas.org) [38] likely obscures subtle, disease‐related changes in blood. Thus, the search for a reliable blood biomarker of TDP‐43 pathology continues, with emerging evidence highlighting cryptic peptides arising from TDP‐43 loss‐of‐function as a potential avenue [39].

Strengths of this study are the inclusion of individuals from the same families with and without pathogenic mutations in the two most common genes causing genetic FTD, combined with the use of an ultrasensitive analytical platform allowing us to accurately measure both established as well as less explored proteins. Limitations include a relatively small sample size, hindering us from finding associations with smaller effect sizes. Inclusion of *MAPT*‐associated FTD alongside *GRN* and *C9orf72* would enable comparison across the three principal genetic subgroups of FTD and help distinguish shared from mutation‐specific proteomic changes. As previous studies have found a signature related to certain lysosomal [5], extracellular matrix, and synapse proteins [6] in *MAPT* mutation carriers, this may have added additional granularity to the present paper. Future studies including *MAPT* carriers will therefore be important to further expand and validate these findings. Finally, it should be noted that LOESS modeling of biomarker relationships with disease severity remains an exploratory cross‐sectional approximation of disease progression and needs to be confirmed using longitudinal data.

## 5 Conclusions

In this exploratory study in plasma, we found changes of both established and previously scantily examined proteins in individuals with genetic FTD. We confirm NfL, and potentially also NfH, as markers of neuroaxonal degeneration in genetic FTD and provide evidence that they may offer complementary information for disease staging, establish GFAP and apoE as indicators of glial responses in GRN‐FTD, and identify NPTXR as a biomarker predominantly altered in symptomatic C9orf72 expansion carriers. The promising findings of this study support the potential to quantify proteins reflecting the complex pathophysiology of genetic FTD if confirmed in future studies, which may be of importance to identify therapeutic targets, track target engagement, and therapeutic response in clinical trials of genetic FTD.

## Author Contributions

**Joel Simrén:** formal analysis, writing – original draft, writing – review and editing, visualization, investigation, software, methodology. **Kübra Tan:** project administration, investigation, writing – review and editing. **Andrea L. Benedet:** conceptualization, writing – original draft, writing – review and editing. **Guglielmo Di Molfetta:** methodology, investigation, writing – review and editing. **Henrik Zetterberg:** conceptualization, writing – review and editing, supervision, funding acquisition, resources. **Ilenia Libri:** writing – review and editing. **Valentina Cantoni:** writing – review and editing. **Barbara Borroni:** conceptualization, writing – review and editing, supervision, funding acquisition. **Nicholas J. Ashton:** conceptualization, writing – original draft, writing – review and editing, supervision, funding acquisition. **Roberta Ghidoni:** writing – review and editing. **Ilaria Pola:** investigation, writing – review and editing. **Jasmine Rivolta:** writing – review and editing.

## Funding

A.L.B. is supported by the Swedish Alzheimer Foundation (grant #AF‐940262), Stiftelsen för Gamla Tjänarinnor, and the Alzheimer's Association Research Fellowship (grant #AARFD‐22‐974564). HZ is a Wallenberg Scholar and a Distinguished Professor at the Swedish Research Council supported by grants from the Swedish Research Council (#2023‐00356, #2022‐01018 and #2019‐02397), the European Union's Horizon Europe research and innovation programme under grant agreement No 101053962, Swedish State Support for Clinical Research (#ALFGBG‐71320), the Alzheimer Drug Discovery Foundation (ADDF), USA (#201809‐2016862), the AD Strategic Fund and the Alzheimer's Association (#ADSF‐21‐831376‐C, #ADSF‐21‐831381‐C, #ADSF‐21‐831377‐C, and #ADSF‐24‐1284328‐C), the European Partnership on Metrology, co‐financed from the European Union's Horizon Europe Research and Innovation Programme and by the Participating States (NEuroBioStand, #22HLT07), the Bluefield Project, Cure Alzheimer's Fund, the Olav Thon Foundation, Beiglers stiftelse, the Erling‐Persson Family Foundation, Familjen Rönströms Stiftelse, Stiftelsen för Gamla Tjänarinnor, Hjärnfonden, Sweden (#FO2022‐0270), the European Union's Horizon 2020 research and innovation programme under the Marie Skłodowska‐Curie grant agreement No 860197 (MIRIADE), the European Union Joint Programme—Neurodegenerative Disease Research (JPND2021‐00694), the National Institute for Health and Care Research University College London Hospitals Biomedical Research Centre, the UK Dementia Research Institute at UCL (UKDRI‐1003), and an anonymous donor.

## Ethics Statement

All participants gave written informed consent according to the Declaration of Helsinki. The local Ethics Committee (NP 2189) approved the study.

## Consent

The authors have nothing to report.

## Conflicts of Interest

J.S. has received speaker honoraria from Roche Diagnostics. H.Z. has served at scientific advisory boards and/or as a consultant for Abbvie, Acumen, Alector, Alzinova, ALZpath, Amylyx, Annexon, Apellis, Artery Therapeutics, AZTherapies, Cognito Therapeutics, CogRx, Denali, Eisai, Enigma, LabCorp, Merry Life, Nervgen, Novo Nordisk, Optoceutics, Passage Bio, Pinteon Therapeutics, Prothena, Quanterix, Red Abbey Labs, reMYND, Roche, Samumed, Siemens Healthineers, Triplet Therapeutics, and Wave, has given lectures sponsored by Alzecure, BioArctic, Biogen, Cellectricon, Fujirebio, Lilly, Novo Nordisk, Roche, and WebMD, and is a co‐founder of Brain Biomarker Solutions in Gothenburg AB (BBS), which is a part of the GU Ventures Incubator Program (outside submitted work). B.B. has served on scientific advisory boards for Alector, Alexion/Astrazeneca, AviadoBio, Lilly, Denali, Wave, UCB. N.J.A. received consultancy or speaker fees from BioArtic, Biogen, Lilly, Quanterix and Alamar Biosciences. The other authors declare no conflicts of interest.

## Supporting information


**Table S1:** List of protein targets in the multiplex panel.
**Table S2:** Raw mean group NPQ values for all analytes in the study.
**Table S3:** Differential abundance results in *GRN*‐FTD vs. non‐carriers.
**Table S4:** Differential abundance results in *C9orf72*‐FTD vs. non‐carriers.
**Table S5:** Differential abundance results in *C9orf72*‐FTD vs. *GRN*‐FTD.
**Table S6:** Differential abundance results in presymptomatic *GRN* carriers vs. non‐carriers.

## Data Availability

The present study includes no data deposited in external repositories. Anonymized data can be shared upon reasonable request from a qualified academic investigator for the sole purpose of replicating procedures and results presented in the article, providing data transfer agrees with EU legislation on General Data Protection Regulation and decisions by institutional review boards.
